# Twelve quick tips for AI-assisted coding in science

**DOI:** 10.1371/journal.pcbi.1014428

**Published:** 2026-07-27

**Authors:** Eric W. Bridgeford, Iain Declan Campbell, Zijiao Chen, Zhicheng Lin, Harrison Ritz, Joachim Vandekerckhove, Russell A. Poldrack

**Affiliations:** 1 Department of Psychology, Stanford University, Stanford, California, United States of America; 2 Princeton Neuroscience Institute, Princeton University, Princeton, New Jersey, United States of America; 3 Department of Psychology, University of Science and Technology of China, Hefei, China; 4 Department of Psychology, Yonsei University, Seoul, Republic of Korea; 5 Department of Cognitive Sciences, University of California, Irvine, California, United States of America; Montreal, CANADA

## Abstract

While AI coding tools have demonstrated potential to accelerate software development, their use in scientific computing raises critical questions about code quality and scientific validity. In this paper, we provide twelve practical tips for AI-assisted coding that balance the capabilities of AI with the demands of scientific and methodological rigor. We address how AI can be leveraged strategically throughout the development cycle with four key themes: problem preparation and understanding, managing context and interaction, testing and validation, and code quality assurance and iterative improvement. These principles serve to emphasize maintaining human agency in coding decisions, establishing robust validation procedures, and preserving the domain expertise essential for methodologically sound research. These tips are intended to help researchers harness AI’s transformative potential for faster software development while ensuring that their code meets the standards of reliability, reproducibility, and scientific validity that research integrity demands.

## 1 Introduction

The integration of artificial intelligence into scientific computing represents one of the most significant shifts in research methodology since the advent of personal computers. Large language models (LLMs) trained on vast corpora of code can now generate syntactically correct, functionally appropriate programs from natural language descriptions, a capability that was inconceivable just a few years ago [1]. Tools like GitHub Copilot, ChatGPT, and Claude have democratized access to sophisticated programming assistance, enabling researchers with limited coding experience to implement complex analyses and build robust scientific software [2]. Agentic coding tools like Claude Code and Cursor have further enabled entire coding workflows by invoking tools outside the language model.

AI-assisted coding tools have shown potential productivity benefits in some controlled studies, with reported gains spanning development speed, code quality, and maintainability [2]. However, the evidence for these benefits remains contested and situation-dependent. While some enterprise studies and developer surveys report significant productivity increases and improved code quality [3], recent randomized controlled trials with experienced developers found that AI tools actually slowed completion times, despite developers believing they were working faster [4]. Additional concerns about code quality have emerged, with research analyzing over 200 million lines of code showing substantial increases in copy-pasted code and decreases in refactoring as AI use has become more prominent [5]. These contradictory findings suggest that productivity effects are far from well-understood, and are likely moderated by developer experience, task complexity, and codebase characteristics. These questions become particularly acute in scientific computing, where code is not merely a means to an end but often embodies scientific reasoning and domain expertise. The validity, reproducibility, and interpretability of scientific software directly impact research integrity and the reliability of scientific findings [6].

The implications for scientific computing are profound. Programming involves complex problem decomposition, algorithmic thinking, and domain-specific reasoning. These are cognitive skills that may atrophy with excessive AI dependence, though the extent of this risk in practice remains an open empirical question. Furthermore, scientific code often requires deep understanding of mathematical models, statistical methods, and domain-specific conventions that cannot be adequately captured by AI tools trained on general programming corpora. These challenges are compounded by the technical limitations inherent to current AI systems: their context windows constrain how much code they can process simultaneously, their stateless nature means they forget previous interactions, and their tendency toward “context rot” [7] can cause them to lose track of important details even within the model’s processing limits.

Effective use of AI coding tools requires understanding how to work within and around these constraints. Techniques like strategic prompting, test-driven development, and externally-managed context files (such as memory files and constitution files) can help maintain consistency across AI interactions [8–10]. Different tools (from conversational interfaces to interactive coding assistants to autonomous coding agents) each offer distinct capabilities and limitations that must be matched to specific development tasks. (For readers unfamiliar with these concepts, we provide detailed definitions in S1 Text.)

The tips presented in this paper emerge from our collective experience using AI-assisted coding tools, and highlight both the substantial promise and documented risks of AI-assisted coding in scientific settings. We hope they provide a framework for harnessing AI’s transformative potential while preserving the methodological rigor and domain expertise essential for high-quality scientific computing. These guidelines emphasize the importance of maintaining human agency in the coding process, establishing robust testing and validation procedures, and strategically managing the interaction between human expertise and AI assistance.

### 1.1 Who is this paper for?

These guidelines are intended for anyone who develops scientific software that will be used more than once, whether by themselves, their collaborators, or the broader research community. This includes both scientists who primarily use code to generate research outputs and developers who build reusable tools and packages. Our focus is on creating maintainable, reliable software rather than one-off scripts. If you write code that needs to work reliably and repeatably, be understood by others, or be built upon in the future, we believe these tips are for you.

Several of the practices that follow may be familiar to readers, including test-driven development, incremental refinement, and structured code review [11–13]. Similarly, many of our recommendations echo best practices for reproducible computational research that predate AI coding tools [6,14,15]. We present them deliberately: scientific software development has historically operated under different constraints than professional software engineering, with researchers often adopting these practices unevenly [16]. AI coding tools change this by substantially lowering the overhead of these practices, making them newly tractable for researchers working without dedicated engineering support. For readers encountering these practices for the first time, we aim to introduce them in the context of AI-assisted workflows; for those already familiar, we aim to clarify how AI changes what they look like in practice.

## 2 The tips

To illustrate these tips in practice with concrete examples, we provide several trivialized examples in S3 Text. These are developed further in an interactive Jupyter Book demonstrating both effective and ineffective implementations of each tip (3+ examples per tip), available at https://poldracklab.org/12qt_ai_assisted_coding and permanently indexed at [17]. We hope these worked examples with detailed analysis will reinforce readers’ understanding of what good and flawed AI interactions look like, and put the strategies discussed herein into practice.

### 2.1 Preparation and understanding

The effectiveness of AI-assisted coding is fundamentally constrained by the clarity and completeness of what you bring to the interaction. AI models cannot compensate for gaps in your understanding of the problem domain, your inability to recognize appropriate solutions, or your unfamiliarity with relevant tools and conventions. These limitations arise because AI tools lack true domain expertise. They pattern-match from training data rather than reason from first principles, and they cannot assess whether their outputs align with field-specific best practices you haven’t mentioned. Without this foundational preparation, you risk “vibe coding,” accepting AI-generated code you cannot evaluate, debug, or maintain [18,19].

#### Tip 1: Gather background knowledge before implementation.

Before writing any code, you need sufficient background knowledge to evaluate whether the code the AI produces is correct, appropriate, and aligned with field conventions. Understand data shapes, missing data patterns, field-specific libraries, and existing implementations that could serve as models. You don’t need to be an expert initially; use AI to help research domain standards, available datasets, common approaches, and implementation patterns before diving into coding. This reconnaissance phase prevents you from reinventing wheels or violating field conventions. Share your current understanding level with the AI and iteratively build context through targeted questions about tools, data structures, and best practices, asking for specific references and paper summaries. This upfront investment ensures that your code aligns with community standards and handles real-world data appropriately.

#### Tip 2: Define your problem and distinguish problem framing from coding.

Framing a problem in a programmatic way and coding are not the same thing [20]. Programmatic problem framing is problem solving: understanding the domain, decomposing complex problems, finding the right levels of abstraction, designing algorithms, and making architectural decisions. Coding is the mechanical translation of these concepts into executable syntax in a programming language. AI tools excel at coding tasks, generating syntactically correct implementations from well-specified requirements, but they currently require human guidance for programmatic problem framing decisions that involve domain expertise, methodological choices, and scientific reasoning. Concretely, this means being able to specify inputs and expected outputs, key constraints and edge cases, and what success looks like before any code is written. You can’t effectively guide or review what you don’t understand, so establish fluency in at least one programming language and fundamental concepts before leveraging AI assistance. This foundation allows you to spot when generated code deviates from best practices or introduces subtle bugs. Without this knowledge, you’re essentially flying blind, unable to distinguish between elegant solutions and convoluted workarounds.

#### Tip 3: Choose appropriate AI interaction models.

It’s tempting to use the AI tools to independently generate a complete codebase, but one quickly ends up being separated from the code and making mistakes. A pair programming model, where one directs interactive AI assistants through comments in the code, can be a way to stay in close touch with the evolution of the codebase. The utility of different language models and tool types will depend on the specific development tasks, developer preferences, and project constraints. A summary of different interaction paradigms, as well as their strengths and limitations in 2025, are provided in Table 1. Because conversational tools, IDE-integrated assistants, and autonomous agents often bundle support for multiple languages and frameworks, performance can vary in ways that are not always apparent from general descriptions or benchmarks. Before committing to a tool, we recommend learning how to properly use the tool and testing it on a representative sample of your own tasks to assess its suitability for your workflow.

**Table 1:** AI-assisted development tools are categorized by interaction model and deployment scenario. Each paradigm offers distinct advantages for different phases of software development, with trade-offs between automation level and developer control. These categories are illustrative: many tools increasingly span paradigms, capabilities evolve rapidly, and some listed tools remain in active development or preview. Examples are not endorsements, and reflect our current understanding of the landscape.

Tool Type	Best For	Description
**Conversational** (ChatGPT, Claude)	Architecture design, complex debugging, learning new concepts	Deep reasoning and flexible problem-solving with extensive context handling, but requires manual code transfer and loses context between sessions
**IDE Assistant** (Copilot)	Code completion, refactoring, maintaining flow	Seamless workflow integration with immediate feedback and preserved code context, but limited reasoning for complex architectural decisions
**Autonomous Agent** (Claude Code, Codex)	Rapid prototyping, multi-file changes, large refactoring	CLI- and API-driven agents that operate across entire codebases with minimal human intervention; many can plug into IDEs as extensions; risk code divergence and require careful monitoring
**Integrated Agentic IDE** (Cursor, Antigravity)	End-to-end feature development, multi-agent workflows, autonomous testing	Full development environments combining editor, agent, and multi-agent orchestration in a single interface, with built-in tools for reviewing and validating agent-generated code

### 2.2 Context engineering and interaction

Once you understand your problem domain and have chosen appropriate tools, effective AI collaboration requires careful management of how you structure prompts and maintain context across interactions. To avoid confusion, we use the word “context” throughout this article only when referring to information that is relevant to the AI system’s operation or your interaction with it. This includes concepts like in-context learning, context management, and context windows, or any general information that is directly relevant to the problem you are trying to solve using AI. Where the word might have otherwise appeared in more general usages (e.g., “in the context of”, “scientific contexts”, etc.) we have used alternate terms for clarity. Most AI systems are stateless, forgetting previous conversations, while others struggle with context rot as conversations grow long. This creates two critical challenges: ensuring the AI has all necessary information to generate appropriate code, and recognizing when a conversation has become too polluted with failed attempts to be productive. This section spans the boundary between preparation and active development: Tip 4 closes out preparation by translating the framed problem into a phased plan, while Tip 5 governs context management within each phase.

#### Tip 4: Think through and divide an implementation plan.

Once you have framed your problem, translate it into a concrete architectural plan before writing any code. Providing the AI with background context (Tip 1) and architectural context (Tip 2), including details about data flow, component interactions, and expected interfaces, transforms it from a code generator into an architecture-aware development partner, generating code that fits naturally into your project rather than producing isolated solutions. Once you have a clear architectural picture, divide the work into discrete, independently testable phases before engaging the AI. Working in phases keeps individual AI sessions focused and short, reducing the risk of context degradation and making it easy to revert to a known-good state if a session goes wrong. You can use LLMs to help generate externally-managed context files, and also look at GitHub Spec Kit for specification-driven workflows that define project requirements and gated phases (Specify, Plan, Tasks). **Go through this entire tip list for each chunk sequentially, avoiding the temptation to implement multiple chunks at once, and regularly cache your results (e.g., via git pushes) as you complete steps.**

#### Tip 5: Manage context strategically.

Context (which for LLMs refers to all of the information currently in the model’s equivalent of “working memory”) is everything in AI-assisted coding. Provide all necessary information upfront for a development phase through clear documentation, attached references, or structured project files with dependencies included. Don’t assume the AI retains perfect context across long conversations; explicitly restate critical requirements, constraints, and dependencies when interactions get complex. Keep track of context and clear or compact when it’s getting close to limits. Use externally-managed context files to keep important context available across sessions while minimizing irrelevant details that can degrade AI performance (see S2 Text). Agents can effectively use these files to keep important things in context for every session. It’s also useful to keep a problem-solving file, where you can add problems whenever you notice them, and where the model can keep track of its progress.

### 2.3 Testing and validation

Testing becomes even more critical when AI generates implementation code. While AI can accelerate code generation, it cannot be trusted to ensure correctness, handle edge cases appropriately, or validate that code meets scientific standards. The tips in this section establish practices for using tests as specifications that guide AI code generation, and for leveraging AI to build and improve comprehensive test coverage.

#### Tip 6: Meticulously verify AI assistance for testing.

A characteristic failure mode of AI-assisted testing is the generation of “paper tests”: models will often modify or generate tests to pass rather than to validate correctness, whether during implementation or when asked to audit coverage. Coding agents may generate placeholder data or mock implementations that merely satisfy the test structure without validating actual logic, inserting fabricated input values or dummy functions that appear to meet acceptance criteria but do not reflect true functionality. These paper tests can be dangerously misleading, seemingly passing while masking broken or incomplete logic. This failure mode arises in two distinct contexts: when the AI is generating implementation code and modifies your tests to pass rather than fixing the underlying code, and when the AI is asked to generate or expand tests directly and produces tests that are structurally valid but logically hollow. In both cases, the defense is the same: always read AI-generated tests carefully and verify that they are testing what you think they are testing, and wherever possible write your own tests before requesting implementation so that the AI must satisfy your criteria rather than criteria it has invented for itself.

#### Tip 7: Use tests to specify and constrain AI-generated code.

AI will respond better to specific test scenarios than vague functionality descriptions. Framing your test requirements as behavioral specifications before requesting implementation forces you to articulate expected inputs/outputs, edge cases, and failure modes upfront [13]. By providing comprehensive test specifications, you guide the AI toward more robust, production-ready implementations. AI tools (such as chatbots or GitHub’s Spec Kit) can help develop these specifications in a way that will optimally guide the model. In addition, whenever a bug is identified during your development cycle, ask the model to generate a test that catches the bug, to ensure that it’s not re-introduced in the future.

#### Tip 8: Leverage AI to audit and expand test coverage.

AI is exceptionally well-suited to auditing your existing coverage: it can systematically enumerate boundary conditions, failure modes, and corner cases that humans tend to overlook. Feed it your function and ask it to generate tests for boundary conditions, type validation, error handling, and numerical stability. Ask it what sorts of problems your code might experience issues with, within your specified API bounds, and why those might (or might not) be relevant to address. AI can help you move beyond testing only expected behavior to robust validation that includes malformed inputs, extreme values, and unexpected conditions. Additionally, you can use AI to review your existing tests and identify gaps in coverage or scenarios you haven’t considered. The AI can help you implement sophisticated testing patterns like parameterized tests, fixtures, and mocking that would be tedious to write from scratch. If you anticipate having future collaborators for your project, you may find it helpful to prioritize building testing infrastructure early. This often includes automated validation workflows, wherein you are able to test your code automatically as you integrate changes into the broader project. AI excels at generating the boilerplate for many of these sophisticated testing tools (such as GitHub Actions, pre-commit hooks, and test orchestration) that ensure your code is validated on every push.

### 2.4 Code quality and validation

Even with comprehensive tests, AI-generated code requires careful human oversight to ensure correctness and appropriateness. AI models can confidently produce code that passes tests but violates domain conventions, introduces subtle bugs, or solves problems in scientifically inappropriate ways. The final tips address critical aspects of quality assurance: actively monitoring AI progress to catch problems early, and critically reviewing and continuing to iterate with all generated code to ensure it meets scientific standards.

#### Tip 9: Monitor progress and know when to restart.

Unlike a human collaborator, an AI agent will not self-flag when it is going in the wrong direction: it will confidently continue making changes, consuming time and tokens, with no indication that it has gone astray. You need to actively monitor what the AI is doing: Is it changing things you didn’t want changed? Is it ignoring the changes you actually requested? Is it introducing new problems while trying to fix old ones? When you notice the AI heading in the wrong direction, stop it rather than letting it continue down an unproductive path. Further, sometimes the most efficient approach is recognizing when a conversation has become too convoluted with failed attempts. When this happens, review your prompt history to identify what went wrong: Were requirements unclear? Did you add conflicting constraints? Did you forget to specify critical details upfront? Starting fresh with these lessons learned often produces better results than continuing to debug within a polluted context. Clear the context and restart from externally-managed context files after adding additional details to prevent the same problem from occurring in the future. This also highlights the need for good version control; if you commit code before undertaking a major change, it’s easy to simply revert to the previous commit and start over if the model goes astray. Fortunately coding agents are generally very good at writing detailed commit messages, making a commit as easy as prompting “commit this to git”.

#### Tip 10: Critically review generated code.

Be skeptical about AI’s claims of success; unlike a human engineer who will typically flag uncertainty or incomplete work, models tend to claim success even when they haven’t really solved the problem. You always need to test the solution independently. Read and understand the code to ensure it solves problems in ways that make sense for your domain and match your prior expectation of how the problem should be solved (e.g., how you anticipated a solution looking based on your pseudocode or architecture schematics you developed in Tip 4). AI-generated code requires careful human review to ensure scientific appropriateness, methodological soundness, and alignment with domain standards. As AI tools accelerate code production, the importance of human peer review has grown rather than diminished: human reviewers often provide qualitatively different and complementary feedback to AI agents, particularly for contextual and domain-specific concerns [21]. Where possible, peer review of AI-generated code by collaborators or lab members therefore provides a valuable additional layer of quality assurance.

#### Tip 11: Refine code incrementally with focused objectives.

Asking an AI to broadly “improve my codebase” is particularly hazardous because the model has no persistent memory of prior architectural decisions: it will make changes that are technically plausible but may silently violate constraints, naming conventions, or design patterns established earlier in development. Instead, approach refinement incrementally with clear, focused objectives. Be explicit about what aspect you want to improve: performance optimization, code readability, error handling, modularity, or adherence to specific design patterns. When you recognize that refinement is needed but can’t articulate the specific approach (for instance, you know certain logic should be extracted into a separate function but aren’t sure how), use AI to help you formulate concrete objectives before implementing changes. Describe what you are trying to achieve and ask the AI to suggest specific refactoring strategies or design patterns that would accomplish your goal, applying the same mindsets delineated in Tips 1–10 to help you along the way.

AI excels at identifying opportunities for refactoring and abstraction, such as recognizing repeated code that should be extracted into reusable functions or methods, and detecting poor programming patterns like deeply nested conditionals, overly long functions, tight coupling between components, and sloppy or inconsistent variable naming conventions. When requesting refinements, specify the goal (e.g., “extract the data validation logic into a separate function” rather than “make this better”) and verify each change against your tests (while expanding your testing as you iterate to reflect updates and improvements) before moving to the next improvement. This focused approach prevents the AI from making changes that, while technically sound, don’t align with your project’s architectural decisions. Note that AI can inadvertently break previously working code or degrade performance while making stylistic improvements. Always test thoroughly after each incremental change, and revert if the “improvement” introduces problems or doesn’t provide clear benefits.

#### Tip 12: Document your code for reuse and publication.

Writing code that works for you today is not the same as writing code that others can understand, run, and build upon. AI tools can accelerate this finalization step substantially. When preparing AI-assisted code for sharing or publication, work through the following checklist:

1. **Document the AI workflow.** Record the AI tool(s) and version(s) used, the general prompting strategy (e.g., whether context files or autonomous agents were employed), and any persistent context files shared with the model. See the Limitations in Section 3 for a conceptual caveat about reproducibility.
2. **Document the code itself.** Every public function and class should have a docstring describing its inputs, outputs, and behavior. Non-obvious implementation choices, especially those that reflect a specific methodological decision, should have inline comments explaining the reasoning.
3. **Specify the environment.** Include a requirements file, environment.yml, or equivalent that pins dependency versions, so that others can reproduce the computational environment your code was developed and tested in.
4. **Provide a README.** Describe what the project does, how to install it, and how to run it. A brief description of the project structure helps new users orient themselves quickly.
5. **Include working examples.** At least one runnable script or notebook should demonstrate typical usage with realistic inputs and show the expected outputs. Examples are often the first thing a new user reaches for, and they serve as an additional integration test.

AI makes it substantially easier to meet these standards than it was previously: generating docstrings, scaffolding READMEs, and producing example notebooks are all tasks where AI excels. This checklist is a starting point; field- and journal-specific requirements may go further, and we recommend consulting relevant guidance such as Nature’s Code and Software Submission Checklist [22] or resources focused on reproducible scientific computing [6,23] where applicable.

## 3 Discussion

This paper presents twelve tips for leveraging AI coding tools effectively in scientific computing while maintaining methodological rigor and code quality. These tips are organized around four key themes: preparation and understanding, context engineering, testing and validation, and code quality assurance. However, we acknowledge a fundamental reality based on our experiences: even when following these tips, flawless start-to-finish interactions are the exception rather than the norm. The value of these tips lies not in guaranteeing immediate success, but in providing a framework that helps you focus on what matters most for successful interactions while also enabling you to quickly diagnose what went wrong when interactions fail, so you can iterate more effectively on your next attempt.

### 3.1 Putting the tips into practice

**Fig 1:**
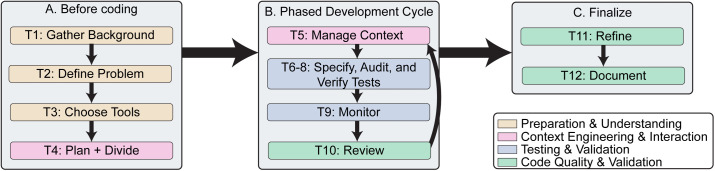
A possible workflow for AI-assisted coding. The twelve tips presented in this paper can be organized into a general interaction flow. Panel A covers preparation before coding (Tips 1–4); Panel B describes the core iterative development loop, which should be repeated for each planned phase (where phases are delineated in tip 4); and Panel C covers finalization and documentation (Tips 11–12). Color indicates the paper’s four organizing themes. This workflow is illustrative; individual projects may require adaptation.

The workflow in Fig 1 illustrates how the twelve tips can be assembled into a coherent development process. Before writing any code, researchers should complete the preparation steps described in Tips 1 through 4: gathering background context, defining the problem clearly, selecting appropriate tools, and dividing the project into manageable phases. These steps are the foundation on which subsequent AI interactions depend. We recommend summarizing your background readings, problem statement, and phased development plan into a single context file, and collecting relevant background documents into a folder that your chosen AI tool can access throughout subsequent steps.

Once preparation is complete, the phased development cycle (Tips 5 through 10) governs the actual coding work. Each phase of the project identified during planning should pass through this loop: managing context at the start of a session (Tip 5), specifying and auditing tests (Tips 6 through 8), monitoring agent behavior during execution (Tip 9), and reviewing outputs critically before moving forward (Tip 10). The back-arrow from Review (T10) to Manage Context (T5) reflects that the loop repeats for each planned phase, and that critical review within a phase may also uncover a need for another pass before moving on.

The finalization steps in Panel C (Tips 11 and 12) apply once a phase or project reaches stability. Refinement (Tip 11) ensures outputs meet scientific and engineering standards, and documentation (Tip 12) prepares the code for sharing or publication. This workflow is intended as a diagnostic scaffold: when an AI-assisted coding session goes wrong, this structure helps identify which step was skipped or needs to be revisited.

### 3.2 Ethical considerations and responsibility

The use of AI-assisted coding raises fundamental questions about scientific accountability. **When code that generates published results is partly AI-generated, who bears responsibility for errors, methodological flaws, or irreproducible outcomes? The answer must be unequivocal: the scientist**. AI tools are instruments, and like any instrument in science, the researcher using them remains fully accountable for validating their outputs and ensuring methodological soundness. This responsibility cannot be delegated to the AI, regardless of how sophisticated the tool or how confident its outputs appear. Researchers must ensure their AI-assisted code is reproducible, well-documented, and scientifically appropriate. When AI generates code that implements a statistical method or analytical pipeline, the researcher must understand that implementation well enough to defend its appropriateness, explain its limitations, and troubleshoot unexpected results. “AI wrote it” is not a valid defense for flawed methodology or incorrect results. Transparency about AI usage in methods sections, while important, does not diminish this responsibility.

Beyond individual accountability, broader ethical concerns demand serious consideration. The environmental costs of training and running large language models are substantial and measurable [24,25]. These systems consume enormous amounts of energy and computational resources, raising questions about the sustainability of widespread AI adoption. Further, intellectual property questions surrounding AI training on open-source code and the ownership of AI-generated code remain legally and ethically unsettled [26–30]. Courts have yet to definitively rule on whether training on copyrighted code constitutes fair use, whether AI-generated code can be copyrighted, and who owns the rights to such code when models have been trained on proprietary or licensed material. These are fundamental ethical and legal challenges that the scientific community must grapple with as AI tools become embedded in research infrastructure. While these complex issues merit a dedicated treatment beyond our scope here, researchers should recognize that using AI coding tools involves participating in systems with significant unresolved ethical dimensions.

Another immediate concern for many researchers involves data privacy and confidentiality. Patient health information, proprietary code, and embargoed results should *never* be submitted to cloud-based AI tools without explicit institutional approval, as inputs may be logged or used for model training [31]. Where such materials are involved, researchers should seek explicit guidance from their institution on data-use policies and the terms of any relevant data sharing agreements before proceeding, as violations can have serious ethical and legal consequences.

### 3.3 Guardrails for autonomous agents

Autonomous coding agents can make extensive changes across a codebase with minimal human intervention, dramatically accelerating development but introducing risks if not properly constrained. The primary danger lies in granting agents too much control without appropriate safeguards. An agent given broad permissions might break existing functionality, introduce security vulnerabilities, or violate architectural principles while reporting success.

We recommend several guardrails. First, be deliberate about what agents can access: restrict file system permissions to the relevant project directory, avoid connecting agents to production systems or sensitive data, and prefer granting the minimum permissions necessary for the task at hand [32]. Second, commit working code before allowing agent changes, enabling easy rollback. Third, learn how to properly configure agents with explicit constraints about what they can modify and what actions require human approval. Fourth, maintain active monitoring rather than allowing unsupervised operation, as discussed in Tip 9. As autonomous agents become more capable, developing clear and safe practices for constraining and monitoring their behavior will become increasingly important for maintaining scientific rigor and system safety.

### 3.4 Limitations and future directions

We acknowledge that we are operating in a rapidly evolving technological landscape. For reference, GPT-3 (2020) had a context window of 2,048 tokens [33], GPT-4 (2023) expanded this to variants with tens of thousands of tokens [34], and current state-of-the-art models like Gemini 2.5 Pro (2025) [35] can operate with context windows of millions of tokens. In light of this rapid evolution, we have intentionally focused on principles and practices that remain relevant across different AI capabilities. We believe our proposed tips emphasize fundamental skills (domain knowledge, problem decomposition, critical review) and strategies (context management, test-driven development, incremental refinement) that apply regardless of specific tools, and thus far have proven useful throughout the evolution of AI models to-date. We have deliberately avoided prescriptive recommendations and strategies tied to specific models, as these would quickly become outdated. Future advances may change which practices prove most valuable, but we believe these tips provide a useful framework for current practice that will remain adaptable as technology matures.

We also anticipate substantial evolution in how scientists acknowledge the role of AI in their work. As AI coding becomes standard practice, we expect clearer community expectations for documenting AI tool usage and validating AI-generated code; this may include citations of specific systems, disclosure of prompting approaches, detailed validation procedures in methods sections, and heightened expectations regarding testing, validation, and reproducibility of code derivatives. A subtlety worth noting is that the AI-assisted process is itself not fully reproducible: AI outputs are stochastic and model versions change over time, so identical prompts may produce different code across sessions. Documentation of the process (as outlined in Tip 12) is therefore a complement to, not a substitute for, archiving and version-controlling the resulting code. The practices we recommend (systematic context building, comprehensive testing, and critical validation) may provide a foundation for informing and meeting these emerging accountability standards.

### 3.5 Further reading

The tips presented in this paper provide a framework for using AI tools effectively in scientific computing, but they build upon foundational concepts in software development, reproducibility, and design. The following resources introduce key concepts and practices that support the development of programming skills necessary for effective AI-assisted coding, and may help readers deepen their understanding of principles underlying the tips.

1. LeVeque, Randall J., et al. Reproducible Research for Scientific Computing: Tools and Strategies. *Computing in Science & Engineering*, vol. 14, no. 4, 2012, pp. 13–17 [11]. This article establishes best practices for reproducible scientific computing that inform our emphasis on documentation, testing, and validation throughout the tips.
2. Ousterhout, John. *A Philosophy of Software Design*. 2nd ed., Yaknyam Press, 2021 [12]. This book articulates core principles of software design, including abstraction and modularity, that inform our guidance in Tips 4 and 5 on specifying context for scientific problems and thinking through incremental refinement in Tip 11.
3. Poldrack, Russell A. *Better Code, Better Science*. https://poldrack.github.io/BetterCodeBetterScience. Accessed Sept 10, 2025 [6]. This comprehensive guide introduces AI tools in scientific workflows and provides practical guidance that complements the principles outlined in our tips.
4. Felleisen, Matthias, et al. *How to Design Programs: An Introduction to Programming and Computing*. 2nd ed., MIT Press, 2018, https://htdp.org [36]. This text emphasizes systematic problem decomposition and design principles that underpin the distinction between problem framing and coding discussed in Tip 2.
5. Beck, Kent. *Test-Driven Development: By Example*. Addison-Wesley, 2003 [13]. This book provides the foundational methodology for test-driven development discussed in Tip 7, demonstrating how writing tests before implementation can help you to develop more robust and maintainable code.
6. Wiebels, Kristina, and David Moreau. Leveraging Containers for Reproducible Psychological Research. *Advances in Methods and Practices in Psychological Science*, vol. 4, no. 2, 2021 [37]. This paper demonstrates how containerization supports reproducible research, directly relevant to the guardrails for autonomous agents discussed in our Section 3.

## Supporting information

S1 Text Background concepts. A glossary of foundational concepts referenced throughout the paper, including large language models, context windows, context rot, in-context learning, prompting, and test-driven development.
(PDF)

S2 Text Sharing context. A discussion of externally-managed context files, including memory files and constitution files, and how they can be used to maintain persistent project information and behavioral constraints across stateless AI interactions.
(PDF)

S3 Text Supplementary examples. A set of trivialized neuroimaging examples illustrating problem-first specification, strategic restart with updated memory files, behavioral specification through tests, and bounded refactoring requests, each annotated with the tips they demonstrate.
(PDF)
