# Supplementary material for: Twelve quick tips for AI-assisted coding in science
Source: PLoS Comput Biol. 2026 Jul 27;22(7):e1014428. doi: 10.1371/journal.pcbi.1014428 (PMC13405308; doi:10.1371/journal.pcbi.1014428)
Supplement: S1 Text — A glossary of foundational concepts referenced throughout the paper, including large language models, context windows, context rot, in-context learning, prompting, and test-driven development. (PDF) [file pcbi.1014428.s001.pdf]

Twelve quick tips for AI-assisted coding in science S1 Text: Background concepts

Eric W. Bridgeford[1,*], Iain Declan Campbell[2], Zijiao Chen[1], Zhicheng Lin[3,4], Harrison Ritz[2], Joachim Vandekerckhove[5], Russell A. Poldrack[1]

1 Department of Psychology, Stanford University, Stanford, California, United States of America
2 Princeton Neuroscience Institute, Princeton University, Princeton, New Jersey, United States of America
3 Department of Psychology, University of Science and Technology of China, Hefei, China
4 Department of Psychology, Yonsei University, Seoul, Republic of Korea
5 Department of Cognitive Sciences, University of California, Irvine, California, United States of America

* Corresponding author: ericwb95@gmail.com

To navigate the challenges of AI-assisted coding effectively, researchers should be familiar with several key concepts that underpin these tools:

- **Large Language Models (LLMs)** are neural networks trained on vast text corpora that generate text by predicting sequences of tokens, basic units of text processing that typically represent words, parts of words, or individual characters [1]. For instance, the word “unhappily” might be tokenized as “un”, “##happi”, “##ly”, where ## marks tokens that are not the start of a word.

- **Context windows** define the maximum number of tokens an LLM can consider when generating responses. State-of-the-art models typically handle hundreds of thousands to millions of tokens, constraining how much code and documentation they can simultaneously process. When context limits are exceeded, models lose track of earlier information. Even when information is contained within the context window, attention to mid-document details can degrade (“lost in the middle”), especially for models with very large context windows; this phenomenon is known as **context rot**. For an example of context rot, see [2].

- **In-context learning** allows models to adapt their behavior based on examples and instructions provided within the current conversation, without permanent changes to the underlying model. This enables direction of model behavior through strategic provision of examples and formatting of instructions.

- **Prompting** encompasses techniques for structuring inputs to elicit desired outputs, including clear requirement specification, strategic provision of examples, and structured formatting. Effective prompting can dramatically improve code quality and relevance.

- **Test-driven development** involves writing tests before implementation to specify expected behavior and validate correctness, a practice that becomes even more critical when AI generates the implementation code. Test driven development is detailed in [3].

References

1. Vaswani A, Shazeer N, Parmar N, Uszkoreit J, Jones L, Gomez AN, et al. Attention is all you need. In: Advances in neural information processing systems. vol. 30; 2017. .

2. Chroma. Context Rot: How Increasing Input Tokens Impacts LLM Performance. Chroma; 2024. Available from: `https://research.trychroma.com/context-rot`.

3. Beck K. Test-Driven Development: By Example. Boston, MA: Addison-Wesley; 2003.
