# Supplementary material for: Twelve quick tips for AI-assisted coding in science
Source: PLoS Comput Biol. 2026 Jul 27;22(7):e1014428. doi: 10.1371/journal.pcbi.1014428 (PMC13405308; doi:10.1371/journal.pcbi.1014428)
Supplement: S2 Text — A discussion of externally-managed context files, including memory files and constitution files, and how they can be used to maintain persistent project information and behavioral constraints across stateless AI interactions. (PDF) [file pcbi.1014428.s002.pdf]

Twelve quick tips for AI-assisted coding in science S2 Text: Sharing context

Eric W. Bridgeford[1,*], Iain Declan Campbell[2], Zijiao Chen[1], Zhicheng Lin[3,4], Harrison Ritz[2], Joachim Vandekerckhove[5], Russell A. Poldrack[1]

1 Department of Psychology, Stanford University, Stanford, California, United States of America
2 Princeton Neuroscience Institute, Princeton University, Princeton, New Jersey, United States of America
3 Department of Psychology, University of Science and Technology of China, Hefei, China
4 Department of Psychology, Yonsei University, Seoul, Republic of Korea
5 Department of Cognitive Sciences, University of California, Irvine, California, United States of America

* Corresponding author: ericwb95@gmail.com

AI coding tools range from conversational interfaces like ChatGPT to interactive assistants like GitHub Copilot to autonomous coding agents like Cursor. Each presents unique challenges for maintaining project context across sessions. Most AI systems are stateless, meaning they forget previous interactions, while others have limited understanding of broader project requirements. This creates two critical problems: context fragmentation, where important project details are lost between sessions, and iteration drift, where AI assistance gradually diverges from intended goals without proper oversight.

Externally-managed context files help address these limitations by providing persistent information across AI interactions. These include:

- **Memory files** contain project-specific information like architectural decisions, software development standards and practices, and lessons learned that persist between interactions. They prevent repetition of past mistakes and ensure each new AI session starts with relevant context. Paolo Perrone [1] provides a detailed description of how to use memory files to improve AI interactions.

- **Constitution files** establish non-negotiable principles governing AI behavior throughout development, such as security requirements or methodological constraints. Kyrychenko et al. [2] details composition of constitution files and how they can be used to contain AI interaction behavior.

Together, these tools can help transform AI interactions into consistent, goal-directed collaboration by providing the persistent context and boundaries that AI systems lack natively.

References

1. Perrone P. The Complete Guide to AI Agent Memory Files (CLAUDE.md, AGENTS.md, and Beyond); 2025. Medium.

2. Kyrychenko Y, Zhou K, Bogucka E, Quercia D. C3AI: Crafting and Evaluating Constitutions for Constitutional AI. In: Proceedings of the ACM on Web Conference 2025. WWW '25. New York, NY, USA: Association for Computing Machinery; 2025. p. 3204–3218. Available from: https://doi.org/10.1145/3696410.3714705.
