# Supplementary material for: Twelve quick tips for AI-assisted coding in science
Source: PLoS Comput Biol. 2026 Jul 27;22(7):e1014428. doi: 10.1371/journal.pcbi.1014428 (PMC13405308; doi:10.1371/journal.pcbi.1014428)
Supplement: S3 Text — A set of trivialized neuroimaging examples illustrating problem-first specification, strategic restart with updated memory files, behavioral specification through tests, and bounded refactoring requests, each annotated with the tips they demonstrate. (PDF) [file pcbi.1014428.s003.pdf]

Twelve quick tips for AI-assisted coding in science S3 Text: Supplementary examples

Eric W. Bridgeford[1,*], Iain Declan Campbell[2], Zijiao Chen[1], Zhicheng Lin[3,4], Harrison Ritz[2], Joachim Vandekerckhove[5], Russell A. Poldrack[1]

1 Department of Psychology, Stanford University, Stanford, CA, USA
2 Princeton Neuroscience Institute, Princeton University, Princeton, NJ, USA
3 Department of Psychology, University of Science and Technology of China, Hefei, China
4 Department of Psychology, Yonsei University, Seoul, Republic of Korea
5 Department of Cognitive Sciences, University of California, Irvine, CA, USA

* Corresponding author: ericwb95@gmail.com

The following examples are intentionally simplified to keep the focus on the interaction pattern being illustrated rather than the complexity of the underlying problem. In practice, the prompts would be longer, the codebases larger, and the bugs more difficult to spot. The neuroimaging domain is used consistently across examples for continuity, but the patterns themselves generalize broadly. Readers are encouraged to consult the accompanying Jupyter Book at https://poldracklab.org/12qt_ai_assisted_coding for more extended examples with detailed analysis of both effective and ineffective interactions [1].

Domain background for non-neuroimaging readers

The examples below use functional MRI (fMRI) data, a neuroimaging modality that measures brain activity indirectly through changes in blood oxygenation. An fMRI dataset is typically organized as a 4D array of shape `(x, y, z, time)`, where the first three dimensions represent spatial location in the brain and the fourth represents measurements taken at successive timepoints (typically one every 1–2 seconds). The brain is parcellated into N regions of interest, and the activity timeseries for each region is summarized as a 1D vector of length T (number of timepoints). *Functional connectivity* refers to the statistical association between pairs of regional timeseries, most commonly measured as Pearson correlation, and is represented as an $N \times N$ symmetric matrix (e.g., a correlation matrix in time). Some timepoints may be excluded from analysis due to excessive head motion (*motion censoring*), leaving missing values (`NaN`) in the timeseries that must be handled carefully to avoid biasing the correlation estimates. Readers unfamiliar with neuroimaging can treat the connectivity matrix as analogous to any pairwise association matrix computed from multivariate timeseries data, such as a gene co-expression matrix in genomics or a sensor correlation matrix in physical measurement systems.

Problem-first specification

This example illustrates a single-turn interaction where the user requests a function to compute a functional connectivity matrix from fMRI timeseries data. The prompt has been refined prior to submission, either through personal editing or preceding turns with the AI. Note the declarative structure: inputs and outputs are defined explicitly,

edge cases (NaN handling, symmetry) are anticipated, and the user provides concrete self-verification criteria. This gives the AI a clear specification to implement against and gives the user a basis for evaluating whether the response is correct.

Relevant Tips: Tip 2 (Define your problem and distinguish problem framing from coding) and Tip 4 (Think through and divide an implementation plan)

User:

```
I need to compute functional connectivity between brain regions from fMRI
timeseries data. Here's what I understand about the problem:

Problem structure:
- Input: NxT array where N=number of regions, T=number of timepoints
- Each row is the BOLD signal timeseries for one region
- Need pairwise correlation between all region pairs
- Output: NxN symmetric correlation matrix

Key considerations:
- Some timeseries may have NaN values (motion-censored timepoints)
- Should use Pearson correlation (standard in field)
- Need to handle the NaN case with pairwise-complete observations
- Diagonal should be 1.0 (region correlated with itself)
- Matrix should be symmetric

What I want:
Create function compute_functional_connectivity(timeseries, method='pearson')
that:
- Takes (n_regions, n_timepoints) array
- Returns (n_regions, n_regions) correlation matrix
- Uses numpy/scipy efficiently (no explicit loops)
- Handles NaN via pairwise deletion
- Includes input validation (correct shape, at least 2 timepoints)

I can verify correctness by checking:
- Output shape matches (n_regions, n_regions)
- Diagonal is all 1.0
- Matrix is symmetric
- Values in [-1, 1]
- Matches manual calculation on small test case
```

AI Response:

```
import numpy as np
from numpy.ma import corrcoef

def compute_functional_connectivity(timeseries, method='pearson'):
    """
    Compute functional connectivity from fMRI timeseries.

    Parameters
    ----------
    timeseries : ndarray, shape (n_regions, n_timepoints)
        BOLD signal timeseries for each region
    method : str
        Correlation method ('pearson' only currently supported)

    Returns
    -------
    connectivity : ndarray, shape (n_regions, n_regions)
```

```
        Functional connectivity matrix

    Raises
    ------
    ValueError
        If timeseries has wrong shape or insufficient timepoints
    """
    if timeseries.ndim != 2:
        raise ValueError(f"Expected 2D array, got {timeseries.ndim}D")

    n_regions, n_timepoints = timeseries.shape
    if n_timepoints < 2:
        raise ValueError(f"Need at least 2 timepoints, got {n_timepoints}")

    if method != 'pearson':
        raise NotImplementedError(f"Method '{method}' not supported")

    # Use masked array to handle NaN with pairwise deletion
    masked_data = np.ma.masked_invalid(timeseries)
    connectivity = np.ma.corrcoef(masked_data)

    # Convert masked array to regular array, filling remaining NaN with 0
    connectivity = np.ma.filled(connectivity, fill_value=0.0)

    return connectivity
```

The response satisfies all of the user's stated verification criteria: the output shape is `(n_regions, n_regions)`, the diagonal is preserved as 1.0, symmetry is guaranteed by the correlation operation, and values are bounded to $[-1, 1]$. Critically, the NaN handling via `np.ma.masked_invalid` directly addresses the motion-censoring requirement, which a vague prompt would not have elicited. Compare this to a prompt such as "compute a brain connectivity matrix from my data," which would likely produce a naïve `np.corrcoef` call with no NaN handling, no input validation, and no docstring.

Strategic restart with updated memory file

This example illustrates a situation where a conversation has become polluted with failed attempts and conflicting requirements. Rather than continuing to patch a broken approach, the user copies the failed conversation into a fresh context window and asks a new agent to diagnose what went wrong. The resulting diagnosis is then used to co-produce a persistent memory file before restarting.

Relevant Tips: Tip 5 (Manage context strategically) and Tip 9 (Monitor Progress and know When to restart)

Original (polluted) conversation:

```
[25 messages of failed attempts to implement a neuroimaging preprocessing
pipeline. The function crashes on real data, requirements were added
incrementally, and the AI has made conflicting changes across the session.]
```

User opens a fresh context window: Here, the user pastes the previous (unsuccessful) conversation directly into a fresh context window, and asks AI to try to help identify what went wrong in the interaction.

```
Here is a previous AI interaction that did not go well. The preprocessing
function failed for the following reasons:
```

```
1. It crashed on 4D fMRI input (the AI assumed 3D data throughout)
2. The output format was incompatible with our existing Pipeline class
3. NaN handling for motion-censored timepoints was never addressed

Could you help me identify what was unclear or missing in my original
specification that led to these problems? Please produce for me a persistent
file that I can use and provide in my future interactions for this
specific problem to avoid these issues in the future.
```

AI Response:

```
Looking at your conversation history, I can identify several specification gaps:

1. Data dimensionality was never stated. You referred to "fMRI data" without
   specifying that it is 4D (x, y, z, time). The AI defaulted to 3D, which
   is a reasonable assumption without further context.

2. The integration requirements were implicit. You mentioned "Pipeline" late
   in the conversation, but never specified its expected input format
   (dict[str, np.ndarray]). The AI could not have known this from context.

3. NaN handling was never included in the original requirements. Motion
   censoring is field-specific knowledge the AI did not apply unprompted.

For your next session, I would recommend specifying upfront:
- Array shape and dimensionality of all inputs
- The exact API of any existing classes this must integrate with
- Any domain-specific data issues (missing values, censored timepoints)
```

The AI's diagnosis can then be used to co-produce a persistent context file with the AI, iterating until the specification is complete. An example `memory.md` produced through this process is shown below:

```
# Neuroimaging Pipeline - Preprocessing Module

INPUT:
- 4D fMRI array of shape (x, y, z, time), dtype float32
- Corresponding 3D brain mask of shape (x, y, z), dtype bool
- Optional motion parameters array of shape (n_timepoints, 6); use
  identity transform if not provided

OUTPUT:
- dict with exactly these keys:
    'bold': 4D np.ndarray (x, y, z, time), motion-corrected
    'motion_params': 2D np.ndarray (n_timepoints, 6)
    'mask': 3D np.ndarray (x, y, z), dtype bool

INTEGRATION:
- Output dict is passed directly to Pipeline.process(dict[str, np.ndarray])
- Pipeline expects exactly the keys above; additional keys will raise KeyError
- DataContainer class (src/data/containers.py) is NOT used in this method

DATA ISSUES TO HANDLE:
- Some timepoints may be NaN (motion-censored); preserve NaN via masked
  arrays rather than filling with zeros, which breaks downstream statistics
- Masks may have different spatial resolution than the BOLD data; resample
  mask to match BOLD dimensions before applying
- Motion parameters may be missing entirely; in this case return identity
```

```
  transforms (zeros for translations, zeros for rotations)

VALIDATION:
- Raise ValueError if input is not 4D
- Raise ValueError if spatial dimensions of mask and BOLD do not match
  after resampling
- Raise ValueError if motion_params is provided but has wrong shape
  (must be n_timepoints x 6)

I can verify correctness by checking:
- Output dict has exactly the three required keys
- bold array preserves NaN at censored timepoints
- mask shape matches bold spatial dimensions (x, y, z)
- motion_params shape is (n_timepoints, 6) in all cases
- Pipeline.process() accepts the output without error
```

User starts a new session with the corrected specification:

```
I need to implement Preprocessor.load_and_prepare() for our neuroimaging
pipeline. A complete specification of my proposed pipeline can be found
in memory.md.

Dependencies available: nibabel, numpy, scipy.ndimage for resampling.
```

Note that the fresh-context diagnostic step produces something qualitatively different from self-review: the new agent has no investment in the failed conversation and can identify specification gaps that are invisible from inside a long, tangled exchange. The memory file then encodes these lessons, so the same problems are addressed proactively in a future session.

Behavioral specification through tests

This example illustrates the difference between requesting implementation without a specification versus providing behavioral tests upfront. In the flawed approach, the user asks for code without defining what success looks like. Tests are added as an afterthought and only verify what the code currently does, rather than what it should do. Bugs are discovered in production and patched without adding regression tests, so the cycle repeats. In the positive approach, the user writes tests that define expected behavior before requesting any implementation, covering the happy path, domain-specific requirements (diagonal preservation), edge cases (negative correlations), and input validation. The AI must satisfy the user's criteria rather than criteria it has invented for itself.

Relevant Tips: Tip 7 (Use Tests to specify and constrain AI-generated code) and Tip 6 (Meticulously verify AI assistance for testing)

Flawed approach — implementation without specification:

```
Create a function to threshold brain connectivity matrices.
```

AI Response:

```
def threshold_connectivity(matrix, threshold=0.5):
    """Threshold connectivity matrix."""
    thresholded = matrix.copy()
    thresholded[matrix < threshold] = 0
    return thresholded
```

User adds tests later:

```python
def test_threshold():
    matrix = np.array([[0.8, 0.3], [0.3, 0.9]])
    result = threshold_connectivity(matrix, threshold=0.5)
    assert result[0, 0] == 0.8
    assert result[0, 1] == 0.0
```

Bugs discovered in production:

```
It's zeroing the diagonal! Brain regions are perfectly correlated with
themselves -- we need to preserve the diagonal.
```

The AI patches the code without adding a regression test for diagonal preservation. A subsequent bug is discovered: negative correlations are being incorrectly zeroed. Another patch is applied without a test. The cycle continues.

Positive approach — behavioral specification through tests:

```
I need a function to threshold brain connectivity matrices. Here is what
success looks like through test cases:
```

```python
def test_basic_thresholding():
    """Values below threshold should be zeroed."""
    matrix = np.array([[0.8, 0.3], [0.3, 0.9]])
    result = threshold_connectivity(matrix, threshold=0.5)
    assert result[0, 0] == 0.8 # Above threshold preserved
    assert result[0, 1] == 0.0 # Below threshold zeroed

def test_preserve_diagonal():
    """Diagonal should always be preserved (self-correlation = 1)."""
    matrix = np.array([[1.0, 0.3], [0.3, 1.0]])
    result = threshold_connectivity(matrix, threshold=0.5)
    assert result[0, 0] == 1.0 # Diagonal preserved regardless of threshold
    assert result[1, 1] == 1.0

def test_negative_correlations():
    """Strong negative correlations should be preserved."""
    matrix = np.array([[1.0, -0.7], [-0.7, 1.0]])
    result = threshold_connectivity(matrix, threshold=0.5)
    assert result[0, 1] == -0.7 # Strong negative preserved
    assert result[1, 0] == -0.7

def test_weak_negative_zeroed():
    """Weak negative correlations should be zeroed."""
    matrix = np.array([[1.0, -0.2], [-0.2, 1.0]])
    result = threshold_connectivity(matrix, threshold=0.5)
    assert result[0, 1] == 0.0

def test_symmetry_preserved():
    """Thresholding should preserve matrix symmetry."""
    matrix = np.array([[1.0, 0.6, 0.3], [0.6, 1.0, 0.7], [0.3, 0.7, 1.0]])
    result = threshold_connectivity(matrix, threshold=0.5)
    assert np.allclose(result, result.T)

def test_input_validation():
    """Should reject invalid inputs."""
    with pytest.raises(ValueError):
        threshold_connectivity(np.array([1, 2, 3])) # Not 2D
    with pytest.raises(ValueError):
```

```
    threshold_connectivity(np.array([[1, 2], [3, 4]])) # Not symmetric
```

```
Now implement threshold_connectivity() to pass all these tests.
```

AI Response:

```python
def threshold_connectivity(matrix, threshold=0.5):
    """
    Threshold connectivity matrix while preserving key properties.

    Parameters
    ----------
    matrix : np.ndarray
        Symmetric connectivity matrix
    threshold : float
        Threshold value (applied to absolute values)

    Returns
    -------
    np.ndarray
        Thresholded matrix with diagonal and symmetry preserved
    """
    if matrix.ndim != 2:
        raise ValueError("Matrix must be 2D")
    if not np.allclose(matrix, matrix.T):
        raise ValueError("Matrix must be symmetric")

    # Threshold by absolute value, preserving sign
    thresholded = matrix.copy()
    mask = np.abs(matrix) < threshold

    # Preserve diagonal regardless of threshold
    np.fill_diagonal(mask, False)
    thresholded[mask] = 0

    return thresholded
```

The specification-first approach produces a qualitatively different result. The diagonal preservation requirement, which the vague prompt missed entirely, is encoded in the tests before any code is written. The negative correlation handling reflects a domain-specific requirement that the AI would not have applied unprompted. Because the user defined success criteria upfront, they can verify the response immediately rather than discovering bugs in production.

AI breaks code during refactoring

This example illustrates the difference between asking the AI to broadly "improve" code versus giving it a specific, bounded refactoring objective. Open-ended improvement requests hand the AI an implicit mandate to make whatever changes it considers beneficial, which can produce plausible-looking code that silently alters behavior. Specific, bounded requests constrain the scope of changes so tightly that unintended modifications have little room to occur.

Relevant Tip: Tip 11 (Refine code incrementally with focused objectives)

Flawed approach — open-ended improvement request:

```
Can you improve and clean up this function?
```

```
def analyze_connectivity(timeseries, density=0.2):
    if timeseries.ndim != 2:
        raise ValueError("Expected 2D array (n_regions, n_timepoints)")
    normed = (timeseries - timeseries.mean(axis=1, keepdims=True))
    normed /= (normed.std(axis=1, keepdims=True) + 1e-8)
    masked = np.ma.masked_invalid(normed)
    connectivity = np.ma.filled(
        np.ma.corrcoef(masked), fill_value=0.0
    )
    threshold_value = np.percentile(
        np.abs(connectivity), 100 * (1 - density)
    )
    thresholded = connectivity.copy()
    thresholded[np.abs(thresholded) < threshold_value] = 0
    np.fill_diagonal(thresholded, 0)
    metrics = compute_graph_metrics(thresholded)
    return metrics
```

AI Response:

```
def analyze_connectivity(timeseries, density=0.2):
    """
    Compute thresholded functional connectivity from fMRI timeseries.
    Simplified to use standard numpy operations for clarity.
    """
    if timeseries.ndim != 2:
        raise ValueError("Expected 2D array (n_regions, n_timepoints)")

    # Normalize timeseries
    normed = (timeseries - timeseries.mean(axis=1, keepdims=True))
    normed /= (normed.std(axis=1, keepdims=True) + 1e-8)

    # Compute correlation matrix (simplified from masked array approach)
    connectivity = np.corrcoef(normed)

    # Threshold and remove self-connections
    threshold_value = np.percentile(
        np.abs(connectivity), 100 * (1 - density)
    )
    thresholded = connectivity.copy()
    thresholded[np.abs(thresholded) < threshold_value] = 0
    np.fill_diagonal(thresholded, 0)

    return compute_graph_metrics(thresholded)
```

The response looks cleaner and the docstring is an improvement. However, the AI has silently replaced the masked array correlation with `np.corrcoef()`, noting in the docstring that it has been "simplified." This is a behavioral change: the original handled NaN values from motion-censored timepoints via pairwise deletion, whereas `np.corrcoef()` will propagate NaN silently through the entire connectivity matrix. The code is superficially better and will pass tests on clean data, making this a particularly dangerous kind of regression.

Positive approach — specific, bounded request:

```
I want to refactor analyze_connectivity() by extracting the correlation
computation into a private method _compute_connectivity(timeseries).
```

```
Constraints:
- Do not change any logic, variable names, or algorithms
- Do not add, remove, or reorder any operations
- The only change should be the extraction itself
- analyze_connectivity() should call _compute_connectivity() and produce
  identical output for all inputs
```

AI Response:

```
def _compute_connectivity(timeseries):
    """Compute correlation matrix with NaN handling via pairwise deletion."""
    masked = np.ma.masked_invalid(timeseries)
    connectivity = np.ma.filled(
        np.ma.corrcoef(masked), fill_value=0.0
    )
    return connectivity

def analyze_connectivity(timeseries, density=0.2):
    """
    Compute thresholded functional connectivity from fMRI timeseries.
    """
    if timeseries.ndim != 2:
        raise ValueError("Expected 2D array (n_regions, n_timepoints)")
    normed = (timeseries - timeseries.mean(axis=1, keepdims=True))
    normed /= (normed.std(axis=1, keepdims=True) + 1e-8)
    connectivity = _compute_connectivity(normed)
    threshold_value = np.percentile(
        np.abs(connectivity), 100 * (1 - density)
    )
    thresholded = connectivity.copy()
    thresholded[np.abs(thresholded) < threshold_value] = 0
    np.fill_diagonal(thresholded, 0)
    return compute_graph_metrics(thresholded)
```

User runs tests:

```
pytest tests/test_connectivity.py -v
# All tests pass
```

The bounded request produces exactly the intended change and nothing else. The NaN handling logic is preserved because the AI was given no mandate to simplify or improve it. This illustrates a general principle: when refactoring AI-assisted code, specify what should change and explicitly state what should not. Phrases like "improve", "clean up", or "simplify" invite the AI to make judgments about what constitutes an improvement, and those judgments may not align with domain-specific requirements the AI is unaware of. The more precisely you define the scope of a refactoring, the less room there is for unintended behavioral changes to slip through.

References

1. Bridgeford E. Poldracklab/12qt_ai_assisted_coding: V0.0. Zenodo; 2025. Available from: https://doi.org/10.5281/zenodo.17398109.
